# Associations between maternal plasma zinc concentrations in late pregnancy and LINE-1 and Alu methylation loci in the young adult offspring

**DOI:** 10.1371/journal.pone.0279630

**Published:** 2022-12-30

**Authors:** Amaraporn Rerkasem, Sothida Nantakool, Brooke C. Wilson, Ampica Mangklabruks, Kongsak Boonyapranai, Apiwat Mutirangura, José G. B. Derraik, Kittipan Rerkasem

**Affiliations:** 1 Environmental—Occupational Health Sciences and Non-Communicable Diseases Research Group, Research Institute for Health Sciences, Chiang Mai University, Chiang Mai, Thailand; 2 Liggins Institute, University of Auckland, Auckland, New Zealand; 3 Department of Internal Medicine, Faculty of Medicine, Chiang Mai University, Chiang Mai, Thailand; 4 Center of Excellence of Molecular Genetics of Cancer and Human Diseases, Department of Anatomy, Faculty of Medicine, Chulalongkorn University, Bangkok, Thailand; 5 Department of Women’s and Children’s Health, Uppsala University, Uppsala, Sweden; 6 Department of Paediatrics: Child and Youth Health, Faculty of Medical and Health Sciences, University of Auckland, Auckland, New Zealand; 7 Clinical Surgical Research Centre, Department of Surgery, Faculty of Medicine, Chiang Mai University, Chiang Mai, Thailand; University of Mississippi Medical Center, UNITED STATES

## Abstract

**Background:**

In animal models, prenatal zinc deficiency induced epigenetic changes in the fetus, but data in humans are lacking. We aimed to examine associations between maternal zinc levels during pregnancy and DNA methylation in LINE-1 and Alu repetitive sequences in young adult offspring, as well as anthropometry and cardiometabolic parameters.

**Methods:**

Participants were 74 pregnant women from the Chiang Mai Low Birth Weight cohort, and their offspring followed up at 20 years of age. Maternal plasma zinc concentrations were measured at approximately 36 weeks of gestation. DNA methylation levels in LINE-1 and Alu repetitive sequences were measured in the offspring, as well as anthropometry and cardiometabolic parameters (lipid profile, blood pressure, and glucose metabolism).

**Results:**

Over half of mothers (39/74; 53%) were zinc deficient (<50 μg/dL) during their third trimester of pregnancy. Maternal zinc concentrations during pregnancy were associated with LINE-1 DNA methylation levels in adult offspring. Specifically, lower prenatal zinc concentrations were associated with: 1) lower levels of total LINE-1 methylation; 2) lower levels of LINE-1 hypermethylation loci; and 3) higher levels of LINE-1 partial methylation loci. Prenatal zinc concentrations were not associated with Alu methylation levels, nor with any anthropometric or cardiometabolic parameters in adult offspring. However, we observed associations between Alu and LINE-1 methylation patterns and cardiometabolic outcomes in offspring, namely total cholesterol levels and diastolic blood pressure, respectively.

**Conclusions:**

Lower maternal zinc concentrations late in gestation were associated with changes in DNA methylation in later life. Thus, zinc deficiency during pregnancy may induce alterations in total LINE-1 methylation and LINE-1 hypermethylation loci. These results suggest a possible epigenetic link between zinc deficiency during pregnancy and long-term outcomes in the offspring.

## Introduction

Zinc is the second most common essential trace element in the human body after iron [1, 2]. Due to its influence on enzyme activity, zinc has several essential roles in protein and nucleic acid metabolism, cellular division, and epigenetic regulation [1, 2]. Zinc is mainly found in seafood, whole grains, beans, red meat, poultry, and dairy products [3]. Its deficiency during pregnancy is a risk factor for several complications, including difficult delivery, fetal malformation, and growth retardation [2]. In addition, maternal zinc deficiency has been linked to adverse health outcomes in the offspring, such as an increased risk of behavioural problems, impaired immune competence, and higher blood pressure in early childhood [4, 5]. A systematic review and meta-analysis of randomized controlled trials showed that zinc supplementation during pregnancy reduces the risk of preterm birth by approximately 14% [6]. As a result, the WHO and UNICEF recommend daily micronutrient supplementation (including zinc, iron, and folic acid) for pregnant women in settings with a high prevalence of malnutrition [7].

There is increasing recognition that a suboptimal maternal nutritional status can adversely impact offspring health as a result of epigenetic modifications [8]. In particular, several animal studies have shown that maternal zinc deficiency alters both DNA methylation and histone acetylation in the fetal epigenome [9–12]. For example, the offspring of mice fed a low-zinc diet during pregnancy had increased DNA methylation and histone acetylation in the promoter region of metallothionein 2 (MT2), a protein involved in zinc transport [10]. These epigenetic modifications led to an increase in MT2 expression in offspring exposed to low zinc levels *in utero* compared to mice whose mothers were fed the control diet [10]. While that study highlights the potential for nutritionally-induced epigenetic modifications in mice, the long-term impacts of maternal zinc deficiency during pregnancy in humans (including the epigenome) have not been explored.

Interspersed repetitive sequences (IRS) are DNA sequences scattered throughout the genome that result from transposition events carried out by transposable elements (TEs) [13]. The retrotransposons LINE-1 and Alu represent two of the most prevalent TEs, constituting up to 17% and 11% of the human genome, respectively [14]. In somatic cells, these retrotransposons are primarily kept in an inactive state through DNA methylation [15]. However, epigenetic modifications such as hypomethylation can sometimes trigger their activation, consequently leading to genomic instability, alterations in gene expression, and the development of disease [16]. For example, LINE-1 and Alu activation is a common hallmark of many human cancers, including colorectal, lung, liver, oesophageal, prostate, and endometrial [17]. LINE-1 and Alu have also been associated with adverse metabolic phenotypes [18], type 2 diabetes [19], and various autoimmune conditions in humans [15, 20, 21].

In Northern Thailand, poverty and maternal undernutrition have been linked to a high incidence of low birth weight compared to other regions in the country [22]. We have previously reported that among children with intrauterine growth restriction, those with subsequent catch-up growth had higher levels of Alu methylation than those who did not exhibit catch-up growth [23]. There is also some evidence that offspring phenotypes associated with maternal undernutrition during pregnancy are driven by modifications to the fetal epigenome [24]. As a result, we hypothesized that maternal plasma zinc levels in late pregnancy are associated with anthropometry and cardiometabolic outcomes in the offspring, and that these associations are influenced by DNA methylation, as evidenced by differential methylation of LINE-1 and Alu elements. Therefore, we examined the associations between maternal zinc levels during pregnancy and DNA methylation in LINE-1 and Alu repetitive sequences in the young adult offspring. In addition, we examined associations between maternal zinc levels and offspring anthropometric and cardiometabolic outcomes.

## Materials and methods

### Ethics

Ethics approval for the study was granted by the Research Ethics Committee at the Research Institute for Health Science, Chiang Mai University (approval number 44/2013). Written informed consent was obtained from all participants.

### Study population

Participants were the offspring of women from the Chiang Mai Low Birth Weight Study (CMLBWS), a hospital-based epidemiological study that examined factors associated with low birth weight in areas with high levels of socioeconomic deprivation in Northern Thailand in 1989–1990 [22]. Pregnant women were enrolled at their first antenatal visit (≤24 weeks of gestation) at the Maharaj Nakorn Chiang Mai Hospital and the Mother and Child Hospital, the two largest providers of antenatal and delivery services in Northern Thailand at the time [22].

Simultaneously, a sub-study was performed on a randomly selected subgroup of women to explore the offspring outcomes associated with plasma zinc concentrations and other trace elements during pregnancy. From this subgroup, women were excluded if they were carrying twins, had an abortion or miscarriage, delivered at other hospitals, or had missing late gestation zinc measurements.

**Fig 1:**
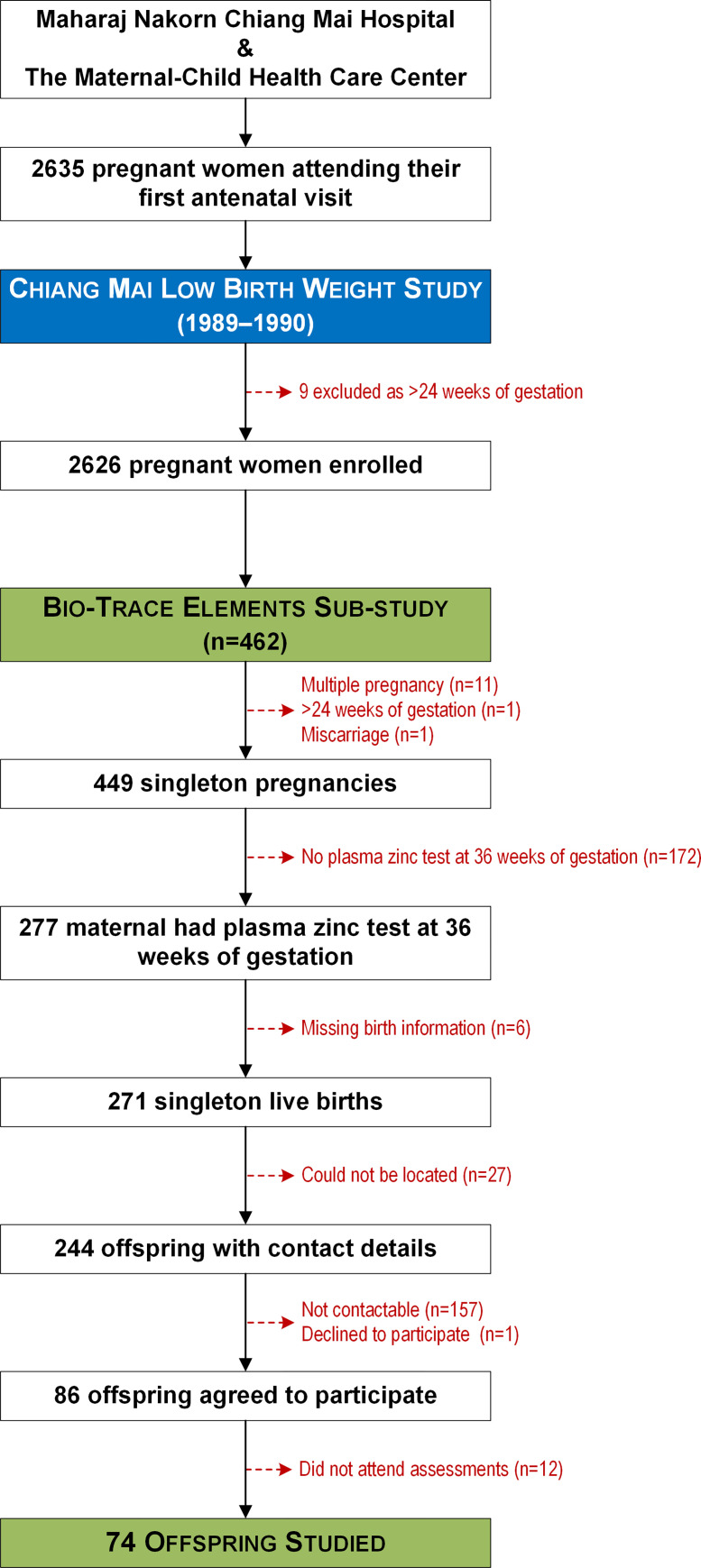
Flow diagram outlining the recruitment of participants into the Chiang Mai Low Birth Weight Study (1989–1990) and subsequently to the follow-up study on the offspring (2010).

In 2010, their young adult offspring were invited to participate in a follow-up study at approximately 20 years of age [23]. Participants whose mothers had taken part in the above-described sub-study were invited to have DNA methylation levels assessed [23]. The study flow diagram is provided as Fig 1.

### Maternal assessments

Mothers were interviewed at their first antenatal visit, when anthropometric and demographic data were collected [22]. At approximately 36 weeks of gestation, fasting whole-blood samples were collected in the morning to measure biochemical parameters, including plasma zinc concentrations. Pregnancy outcomes and birth parameters were subsequently measured as previously described [22, 25]. Birth weights and lengths were converted into *z*-scores per INTERGROWTH-21^st^ standards [26].

Maternal plasma samples were obtained following whole blood centrifugation at 2500 rpm for 10 minutes and stored long-term at -20°C. Zinc concentrations were measured at the Research Institute for Health Sciences (RIHES), Chiang Mai University using atomic absorption spectrometry [27].

### Offspring assessments

The offspring who could be located and consented to participate underwent clinical assessments at RIHES, following a 12-hour overnight fast. Demographic data were collected using questionnaires. Clinical assessments included anthropometry [height, weight, and body mass index (BMI)], blood pressure, and lipid profile [high-density lipoprotein cholesterol (HDL) and low-density lipoprotein cholesterol (LDL), triglycerides, and total cholesterol], as previously described [25]. Fasting glucose and insulin levels were measured, and the homeostatic model assessment of insulin resistance (HOMA-IR) was calculated [28]. In addition, blood samples were used for DNA methylation assessment.

### DNA methylation assessment

The detailed methodology for the DNA methylation analysis has been previously reported [23, 29]. The Combined Bisulfite Restriction Analysis (qCOBRA) method was used to quantify both global and locus-specific DNA methylation with respect to LINE-1 and Alu repetitive elements. In brief, DNA was extracted from peripheral blood mononuclear cells using the phenol-chloroform extraction method. The EZ DNA methylation-Gold Kit ^TM^ (Zymo research corp, Orange, CA, USA) was used for denaturation and bisulfite conversion following the manufacturer’s instructions. Bisulfite-treated DNA was subsequently amplified for 40 cycles with primers specific for LINE-1 (Fwd 5’-CGTAAGGGG TTAGGGAGTTTTT-3’; Rev 5’-RTAAAACCCTCCRAACCAAATATAAA-3’; annealing temperature 50°C) and Alu (Fwd 5’-GGCGCGGTGGTTTACGTTTGTAA-3’; Rev 5’TTAATAAAAACGAAAT TTCACCATATTAACCAAAC-3’, annealing temperature 53°C). PCR amplicons were incubated overnight with *Taql* and *Tasl* restriction enzymes in NEBuffer 3.0 (New England Biolabs, Ontario, Canada). The resulting LINE-1 (160bp) and Alu (117bp) fragments were analyzed by electrophoresis on an 8% non-denaturing polyacrylamide gel (Gelstar^TM^, Lonza, Rockland, ME, USA). Daudi, Jurkat, and HeLa cell lines were used as positive controls. Distilled water was used as a negative control.

DNA methylation levels were reported as percentages describing four distinct methylation patterns, as defined by the methylation status at two CpG dinucleotides [23, 29]:

1. Hypermethylation loci containing two methylated CpG (mCmC)
2. Hypomethylation loci containing two unmethylated CpG (uCuC)
3. Partial methylation loci including 5’methylated with 3’unmethylated (mCuC)
4. Partial methylation loci including 5’unmethylated with 3’methylated (uCmC)

### Statistical analyses

Study participants (mothers) were compared to those lost to follow-up with one-way ANOVA or Fisher’s exact tests, as appropriate. Linear associations between plasma zinc concentrations and study outcomes were initially assessed using Pearson’s correlation coefficients, with zinc levels log-transformed to approximate a normal distribution.

For birth outcomes, general linear models were run adjusting for maternal age at baseline and offspring sex, with additional confounders added as follows: maternal BMI for gestational age; maternal BMI [30] and gestational age for birth weight; maternal BMI [30] for birth weight *z*-score; maternal height and gestational age for birth length; and maternal height for birth length *z*-score.

For outcomes at the 20-year follow-up, general linear models were adjusted for maternal age at baseline, gestational age at birth [31], and offspring sex. Models examining potential associations with anthropometry and cardiometabolic outcomes included additional independent variables where appropriate: maternal BMI for offspring weight and BMI [32]; maternal height for offspring height [33]; and pregnancy-induced hypertension [34] and current smoking status for blood pressure [35].

Stratified analyses were subsequently carried out: 1) with mothers categorized as zinc deficient (plasma concentrations <50 μg/dL) [36] or not deficient (≥50 μg/dL); and 2) with mothers stratified into zinc-concentration tertiles. Univariable comparisons were carried out with one-way ANOVA, with general linear models carried out as described above.

Multivariable linear associations are reported as adjusted β coefficients and respective 95% confidence intervals (CI). Group data are reported as means ± standard deviations (SD), and between-group comparisons as adjusted mean differences (aMD) and 95% CI. Data were analysed using SAS v9.4 (SAS Institute, Cary, NC, USA). All statistical tests were two-tailed, with statistical significance maintained at p<0.05 without adjustment for multiple comparisons as per Rothman (1990) [37] to minimize the likelihood of type 2 (false negative) errors due to the exploratory nature of our study and the many non-independent parameters examined. Further, in this context, all statistically significant associations were interpreted with caution.

## Results

### Study population

We studied 74 young Thai adults (43% males) at a mean age of 20.6 years (Table 1). The characteristics of study participants and those lost to follow-up are provided in Table 2; the two groups were largely similar, except our participants included a lower proportion of males and mothers who were approximately 1.3 years older at study entry to the original study.

**Table 1:** Demographic and birth characteristics of the 74 follow-up study participants.

**Birth**	**Sex (males)**	32 (43%)
	**Gestational age (weeks)**	39.0 ± 1.6
	**Caesarean delivery**	5 (7%)
	**Preterm birth**	7 (9%)
	**Birth weight (g)**	2765 ± 359
	**Birth weight z-score**	-1.01 ± 0.82
	**Birth length (cm)**	48.0 ± 2.1
	**Birth length z-score**	-0.52 ± 1.2
	**Low birth weight (<2,500g)**	13 (18%)
	**Placental weight (g)**	523 ± 102
**Follow-up**	**Age (years)**	20.6 ± 0.5
	**Smoking status**	
	Non-smoker	61 (92%)
	Smoker	5 (8%)
	**Education** ^**a**^	
	Less than high school	9 (15%)
	High school or higher	52 (85%)

Data are means ± standard deviation (SD) or n (%), as appropriate.

None of their mothers reported using tobacco or illicit drugs during pregnancy.

^a^ Smoking and education data were missing for 8 (11%) and 13 (18%) participants, respectively.

**Table 2:** Comparison of demographic and birth characteristics between our follow-up study participants and those lost to follow-up.

	Characteristics	Study participants	Lost to follow-up	*P*-value
***n***		74	197	
**Mother**	**Age at baseline (years)** ^**a**^	26.2 ± 4.4	24.9 ± 4.3	**0.030**
	**Body mass index at baseline (kg/m**^**2**^**)** ^**a**^	21.47 ± 2.65	21.16 ± 2.46	0.36
	**Smoking during pregnancy**	nil	4 (2%)	0.22
	**Alcohol consumption during pregnancy**	1 (1%)	2 (1%)	0.81
	**Nulliparous**	50 (68%)	130 (66%)	0.89
	**Pregnancy-induced hypertension** ^**b**^	5 (7%)	7 (4%)	0.32
	**Caesarean delivery**	5 (7%)	28 (14%)	0.14
	**Education level**			
	Less than high school	65 (86%)	162 (82%)	0.47
	High school or above	4 (14%)	35 (18%)	
	**Plasma zinc (μg/dL)** ^**c**^	53.6 ± 25.9	59.1 ± 28.8	0.16
	**Zinc deficiency (<50 μg/dL)** ^**c**^	39 (53%)	89 (45%)	0.27
**Family**	**Spouse/partner education level**			
	Less than high school	51 (68.9%)	128 (65%)	0.54
	High school or above	23 (31.1%)	69 (35%)	
	**Household income (baht/month)** ^**d**^	2500 [2800]	3000 [2200]	0.63
**Offspring**	**Sex (males)**	32 (44%)	120 (61%)	**0.013**

Data are mean ± standard deviation (SD), median [interquartile range], or n (%), as appropriate.

^a^ Recorded at the first antenatal visit (≤24 weeks of gestation).

^b^ Defined as systolic blood pressure ≥140 mmHg and/or diastolic blood pressure ≥90 mmHg during pregnancy, which was developed after 20 weeks of gestation without proteinuria in a woman previously normotensive.

^c^ Measured in late pregnancy at approximately 36 weeks of gestation.

^d^ Income at recruitment to the original study in 1989–1990, unadjusted for inflation.

*P*-values for statistically significant differences (at p<0.05) are shown in bold.

### Linear associations with prenatal zinc concentrations

At birth, after adjustment for confounders, reductions in maternal zinc levels were associated with decreasing birth weight (p = 0.044), weight *z*-score (p = 0.030), and length *z*-score (p = 0.049) (Table 3).

**Table 3:** Linear associations between maternal plasma zinc concentrations in late pregnancy and offspring characteristics at birth.

	Simple linear correlation		Multivariable model	
Parameters	*r*	*p-*value	Adjusted β	*p-*value
**Gestational age (days)**	-0.21	0.08	-0.5 (-1.0, 0.1)	0.11
**Birth weight (g)**	0.09	0.46	17 (1, 33)	**0.044**
**Birth weight z-score**	0.20	0.08	0.047 (0.005, 0.089)	**0.030**
**Birth length (cm)**	0.17	0.16	0.1 (0.0, 0.2)	0.08
**Birth length z-score**	0.26	**0.033**	0.063 (0.000, 0.126)	**0.049**

Data are the Pearson’s correlation coefficients (*r*) and respective p-values; or the adjusted β coefficients and 95% confidence intervals from general linear models adjusting for sex and maternal age at baseline, as well as: maternal body mass index for gestational age; maternal body mass index and gestational age for birth weight; maternal body mass index for birth weight *z*-score; maternal height and gestational age for birth length; and maternal height for birth length *z*-score. Note that maternal zinc data were log-transformed for all analyses; thus, the back-transformed adjusted β coefficients represent the corresponding change in outcome units for every 10% increase in maternal zinc levels. *P*-values for statistically significant associations (at p<0.05) are shown in bold.

**Fig 2:**
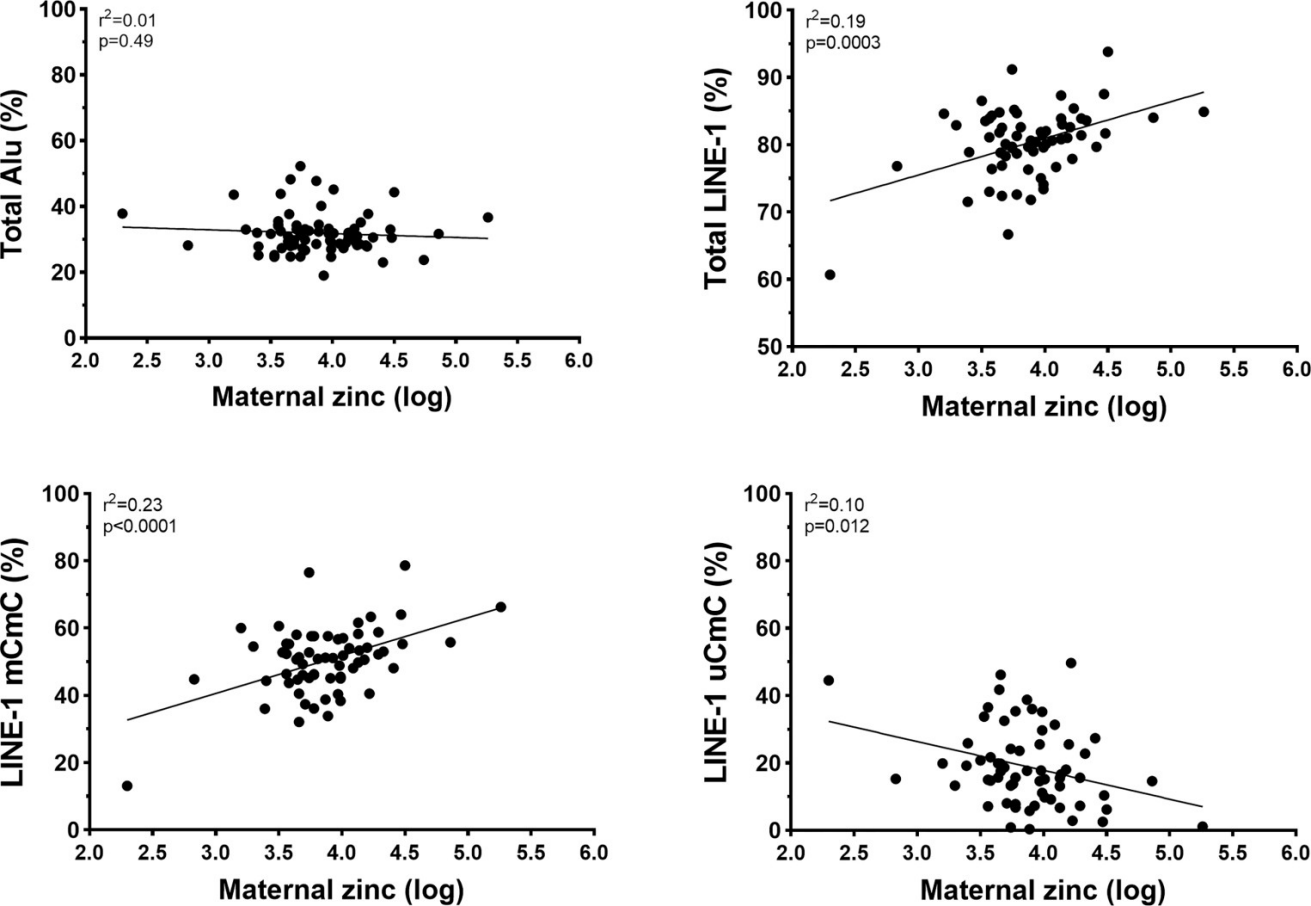
Scatter plots showing linear associations between maternal zinc concentrations in late pregnancy (log-transformed) and methylation levels for total Alu, total LINE-1, LINE-1 mCmC (hypermethylation), and LINE-1 uCmC (partial methylation).

At the 20-year follow-up, in both unadjusted and adjusted analyses, lower zinc concentrations in maternal plasma were associated with decreasing methylation levels of total LINE-1 and LINE-1 hypermethylation loci (mCmC) but increasing LINE-1 uCmC (partial methylation loci) levels in the offspring (Table 4; Fig 2). Conversely, maternal zinc concentrations were not associated with methylation levels of any Alu loci (Table 4; Fig 2) or any anthropometric or cardiometabolic parameters (Table 5).

**Table 4:** Associations between maternal plasma zinc concentrations in late pregnancy and DNA methylation levels in the young adult offspring.

		Simple linear correlation		Multivariable model	
Offspring parameters	*n*	*r*	*p-*value	Adjusted β	*p-*value
**Total Alu (%)**	72	-0.08	0.49	-0.11 (-0.43, 0.22)	0.51
**Alu mCmC (%)**	72	0.01	0.94	0.05 (-0.32, 0.43)	0.77
**Alu mCuC (%)**	72	-0.09	0.47	-0.13 (-0.40, 0.14)	0.33
**Alu uCmC (%)**	72	-0.14	0.25	-0.18 (-0.49, 0.12)	0.23
**Alu uCuC (%)**	72	0.14	0.25	0.27 (-0.16, 0.69)	0.21
**Total Line-1 (%)**	64	0.44	**<0.001**	0.53 (0.28, 0.79)	**<0.001**
**LINE-1 mCmC (%)**	64	0.48	**<0.0001**	1.12 (0.62, 1.63)	**<0.0001**
**LINE-1 mCuC (%)**	64	-0.18	0.16	-0.41 (-0.90, 0.07)	0.09
**LINE-1 uCmC (%)**	64	-0.31	**0.012**	-0.80 (-1.46, -0.14)	**0.019**
**LINE-1 uCuC (%)**	64	0.07	0.58	0.09 (-0.21, 0.40)	0.55

Data are the Pearson’s correlation coefficients (*r*) and respective p-values; or the adjusted β coefficients and 95% confidence intervals from general linear models adjusting for sex, maternal age at baseline, and gestational age. Note that zinc data were log-transformed for analyses; thus, the back-transformed adjusted β coefficients represent the change in percentage points in methylation levels for every 10% increase in maternal zinc levels. *P*-values for statistically significant associations (at p<0.05) are shown in bold.

**Table 5:** Associations between maternal plasma zinc concentrations in late pregnancy and anthropometric and cardiometabolic outcomes in the young adult offspring.

			Simple linear correlation		Multivariable model	
	Parameters	*n*	*r*	*p-*value	Adjusted β	*p-*value
**Anthropometry**	**Height (cm)**	74	0.16	0.16	0.1 (-0.2, 0.3)	0.71
	**Weight (kg)**	74	0.06	0.60	0.01 (-0.55, 0.57)	0.97
	**BMI (kg/m** ^**2**^ **)**	74	-0.01	0.95	-0.03 (-0.22, 0.17)	0.78
**Lipid profile**	**Total cholesterol (mg/dL)**	74	-0.02	0.86	-0.2 (-2.3, 2.0)	0.88
	**HDL-C (mg/dL)**	74	-0.07	0.54	0.0 (-0.7, 0.6)	0.96
	**LDL-C (mg/dL)**	74	-0.01	0.94	-0.2 (-2.0, 1.6)	0.81
	**Triglycerides (mg/dL)**	74	0.01	0.80	0.2 (-2.3, 2.7)	0.87
**Blood pressure**	**Systolic (mmHg)**	74	0.03	0.77	0.2 (-0.4, 0.7)	0.57
	**Diastolic (mmHg)**	74	-0.03	0.80	0.0 (-0.6, 0.6)	0.93
**Glucose metabolism**	**Fasting glucose (mg/dL)**	74	-0.07	0.59	-0.19 (-0.57, 0.20)	0.33
	**HOMA-IR**	74	0.06	0.73	-0.01 (-0.10, 0.08)	0.85

Data are the Pearson’s correlation coefficients (*r*) and respective p-values; or the adjusted β coefficients and 95% confidence intervals from general linear models adjusting for sex, maternal age at baseline, and gestational age, with other independent variables added where appropriate: maternal BMI for offspring weight and BMI; maternal height for offspring height; and pregnancy-induced hypertension and current smoking status for blood pressure. Note that zinc data were log-transformed for analyses; thus, the back-transformed adjusted β coefficients represent the change in units of the outcome for every 10% increase in maternal zinc levels.

### Associations stratified by prenatal/maternal zinc status

There was a high incidence of zinc deficiency among mothers, with 53% (n = 39) having plasma concentrations <50 μg/dL (Table 6; S1A Fig). Demographic characteristics in the two groups were similar, except that babies born to mothers with zinc deficiency were shorter by 0.43 standard deviations (Table 6). Offspring methylation levels were also largely similar in the two groups, but lower LINE-1 mCmC levels (-5.1%; p = 0.049) were observed in the offspring of mothers with zinc deficiency (Table 7).

**Table 6:** Demographic characteristics of study participants according to maternal zinc status in late pregnancy.

	Characteristics	Zinc deficient	Not zinc deficient	*P*-value
**n**		39	35	
**Mother (at baseline)**	**Age (years)**	26.4 ± 5.1	25.9 ± 3.6	0.45
	**Maternal body mass index (kg/m** ^**2**^ **)**	21.9 ± 2.9	21.0 ± 2.3	0.38
	**Nulliparous**	24 (62%)	26 (74%)	0.32
	**Pregnancy-induced hypertension** ^**a**^	4 (10%)	1 (3%)	0.36
	**Education level**			
	Less than high school	35 (95%)	30 (94%)	>0.99
	High school or higher	2 (5%)	2 (6%)	
	**Household income (baht/month)** ^**b**^	2500 [1800, 4350]	2500 [1350, 4625]	0.99
**Pregnancy outcomes**	**Sex (male)**	17 (49%)	15 (38%)	0.48
	**Caesarean delivery**	4 (10%)	1 (3%)	0.36
	**Gestational age (weeks)**	39.3 ± 1.6	38.7 ± 1.5	0.15
	**Preterm birth** ^**c**^	4 (10%)	3 (9%)	>0.99
	**Birth weight (g)**	2756 ± 378	2775 ± 343	0.48
	**Birth weight *z*-score**	-1.11 ± 0.85	-0.89 ± 0.79	0.10
	**Low birth weight (<2500g)**	8 (21%)	5 (14%)	0.55
	**Birth length (cm)**	47.7 ± 2.1	48.2 ± 2.2	0.15
	**Birth length *z*-score**	-0.73 ± 1.17	-0.29 ± 1.14	**0.041**
**Offspring (at follow-up)**	**Age (years)**	20.7 ± 0.4	20.4 ± 0.5	**0.026**
	**Body mass index (kg/m** ^**2**^ **)**	20.81 ± 4.14	20.88 ± 3.12	0.34
	**Smoking status**			
	Non-smoker	36 (97%)	25 (86%)	0.16
	Smoker	1 (3%)	4 (13%)	
	**Education of offspring**			
	Less than high school	4 (13%)	5 (17%)	0.72
	High school or higher	28 (88%)	24 (83%)	

Zinc deficiency was defined as a plasma concentration <50 μg/dL. Data are means ± standard deviation (SD), medians [quartile 1, quartile 3], or n (%), as appropriate. *P*-values for statistically significant differences between groups (at *p*<0.05) are shown in bold. There was no reported use of tobacco or illicit drugs during pregnancy.

^a^ Pregnancy-induced hypertension was defined as systolic blood pressure ≥140 mmHg and/or diastolic blood pressure ≥90 mmHg during pregnancy, which was developed after 20 weeks of gestation without proteinuria in a woman previously normotensive.

^b^ Income at recruitment to the original study in 1989–1990, unadjusted for inflation.

^c^ Defined as gestational age at birth <37 weeks.

**Table 7:** DNA methylation levels in young adult offspring according to maternal zinc deficiency in late pregnancy.

	Zinc deficiency	No zinc deficiency	aMD	*P*-value
**n**	39	35		
**Total Alu (%)**	32.7 ± 6.8	31 ± 5.4	1.6 (-1.3, 4.5)	0.29
**Alu mCmC (%)**	10.8 ± 8.1	9.7 ± 5	0.8 (-2.6, 4.1)	0.64
**Alu mCuC (%)**	23.7 ± 6.6	22.2 ± 4.8	0.7 (-1.7, 3.1)	0.56
**Alu uCmC (%)**	20.8 ± 4.9	20.3 ± 4.9	1.3 (-1.4, 4.1)	0.34
**Alu uCuC (%)**	45 ± 8.4	47.7 ± 7.8	-2.6 (-6.4, 1.2)	0.18
**n**	35	29		
**Total LINE-1 (%)**	79.2 ± 6	81.6 ± 4.2	-2.2 (-4.8, 0.3)	0.09
**LINE-1 mCmC (%)**	48 ± 10.9	53.3 ± 8.5	-5.1 (-10.1, 0.0)	**0.049**
**LINE-1 mCuC (%)**	20.3 ± 12	17.2 ± 11.5	2.9 (-1.5, 7.3)	0.19
**LINE-1 uCmC (%)**	23.1 ± 10.3	20.4 ± 6.6	2.9 (-3.3, 9.0)	0.35
**LINE-1 uCuC (%)**	8.5 ± 6.1	9.1 ± 5.1	-0.7 (-3.4, 2.0)	0.61

Zinc deficiency was defined as a plasma level <50 μg/dL in late pregnancy.

Group data are means ± standard deviation; aMD are the adjusted mean differences and respective 95% confidence intervals derived from general linear models, adjusting for sex, maternal age at baseline, and gestational age. *P*-values for statistically significant differences between groups (at *p*<0.05) are shown in bold.

CpG methylation patterns include mCmC, hypermethylation; uCuC, hypomethylation; mCuC and uCmC, partial methylation.

However, the stratification of participants into tertiles (S1B Fig) identified differences in LINE-1 methylation levels between the offspring of mothers in the Higher zinc tertile compared to the other tertiles, in both unadjusted and adjusted analyses (Table 8). Total LINE-1 methylation levels were 4.0 (*p* = 0.014) and 3.2 (*p* = 0.041) percentage points higher in the Higher tertile group compared with the Lower and Mid tertiles, respectively (Table 8). Further, differences of a greater magnitude were observed in the Higher tertile for LINE-1 mCmC compared to the Lower group (+8.7%; *p* = 0.007), with a similar trend compared to the Mid tertile (+6.0%; *p* = 0.052) (Table 8). Conversely, compared to the Lower tertile group, LINE-1 uCmC methylation levels were -7.6 percentage points lower (*p* = 0.048) in the Higher group, with a similar trend for the Mid group (-6.4%; *p* = 0.078) (Table 8). There were no differences in Alu methylation levels among groups (Table 8) whose demographic characteristics were also similar (S1 Table).

**Table 8:** DNA methylation levels in young adult offspring according to tertiles of maternal plasma zinc concentrations in late pregnancy.

	Lower Tertile	Mid Tertile	Higher Tertile	aMD	aMD	aMD
				Higher vs Lower	Higher vs Mid	Mid vs Lower
**n**	24	26	24			
**Total Alu (%)**	32.3 ± 6.3	32.4 ± 7.4	31.0 ± 4.6	-1.1 (-4.7, 2.4)	-0.3 (-3.9, 3.3)	-0.9 (-4.5, 2.8)
**Alu mCmC (%)**	9.9 ± 7.2	11.1 ± 7.9	9.9 ± 5.2	0.3 (-3.8, 4.4)	-0.9 (-5.1, 3.3)	1.2 (-3.0, 5.5)
**Alu mCuC (%)**	21.0 ± 5.1	20.2 ± 4.9	20.6 ± 4.8	-0.6 (-3.5, 2.4)	0.6 (-2.4, 3.6)	-1.2 (-4.2, 1.8)
**Alu uCmC (%)**	23.9 ± 6.2	23.3 ± 6.9	21.7 ± 3.9	-2.1 (-5.5, 1.3)	-0.6 (-4.0, 2.8)	-1.5 (-4.9, 1.9)
**Alu uCuC (%)**	45.3 ± 8.5	45.9 ± 9.4	47.8 ± 6.3	2.5 (-2.2, 7.2)	0.3 (-4.4, 5.0)	2.2 (-2.5, 7.0)
**n**	21	25	18			
**Total LINE-1 (%)**	79.0 ± 6.0	79.1 ± 5.0	83.3 ± 3.9	4.0 (0.8, 7.1)*	3.2 (0.1, 6.3)*	0.8 (-2.2, 3.7)
**LINE-1 mCmC (%)**	47.2 ± 10.8	48.9 ± 9.3	56.2 ± 8.6	8.7 (2.5, 14.9)**	6.0 (0.0, 12.1)	2.7 (-3.2, 8.5)
**LINE-1 mCuC (%)**	21.2 ± 9.5	23.9 ± 9.4	20.0 ± 6.9	-1.4 (-7.0, 4.2)	-3.3 (-8.7, 2.2)	1.9 (-3.4, 7.2)
**LINE-1 uCmC (%)**	23.8 ± 11.0	16.9 ± 11.3	16.0 ± 12.2	-7.6 (-15.2, -0.1)*	-1.2 (-8.6, 6.2)	-6.4 (-13.5, 0.7)
**LINE-1 uCuC (%)**	7.8 ± 5.1	10.3 ± 6.6	7.9 ± 4.5	0.3 (-3.1, 3.7)	-1.5 (-4.8, 1.8)	1.8 (-1.4, 5.0)

Tertiles were stratified according to maternal zinc concentrations in plasma in late pregnancy as Lower (<41.0 μg/dL), Mid (≥41.0 but <58.05 μg/dL), and Higher (≥58.05 μg/dL).

Group data are means ± standard deviation; aMD are the adjusted mean differences and respective 95% confidence intervals derived from general linear models, adjusting for sex, maternal age at baseline, and gestational age, with *p<0.05 and **p<0.01 for the pairwise differences between group.

CpG methylation patterns include mCmC, hypermethylation; uCuC, hypomethylation; mCuC and uCmC, partial methylation.

### Associations between LINE-1 and Alu methylation and anthropometric and cardiometabolic outcomes

We observed no associations between the overall LINE-1 and Alu methylation levels and any anthropometric or cardiometabolic outcomes in offspring (S2 and S3 Tables). However, we did identify three associations between specific methylation patterns and cardiometabolic outcomes. LINE-1 partial methylation (uCmC) levels were associated with higher diastolic blood pressure (+2.4 mmHg for every 10% increase; *p* = 0.025) (S2 Table). Alu partial methylation (uCmC) levels were associated with higher cholesterol levels (20.6 mg/dl for every 10% increase; *p* = 0.015) but Alu hypomethylation (uCuC) levels with lower cholesterol levels (-13.6 mg/dl for every 10% increase; *p* = 0.030) (S3 Table).

## Discussion

By following up offspring from the Chiang Mai Low Birth Weight Study, we showed that maternal zinc concentrations during pregnancy were associated with DNA methylation levels in young adult offspring. In particular, lower levels of maternal zinc at the end of pregnancy were associated with lower total LINE-1 methylation and LINE-1 hypermethylation loci. These data are particularly important given the incidence of zinc deficiency observed in this cohort, and the emerging evidence from animal studies that DNA methylation modifications induced during gestation can affect long-term health outcomes in offspring [9, 10].

There was a high incidence of zinc deficiency (53%) in this subgroup of women from the original Chiang Mai Low Birth Weight Study. In the 1990s, the global prevalence of zinc deficiency was estimated at 31%, ranging between 4% and 73% [36]. A low dietary intake of zinc remains relatively common in rural Thailand, where edible plants rich with phytates and glutinous rice cultivated in low-zinc soils remain the mainstay of their diet [38]. It has been estimated that people in Thailand have a "medium" risk of zinc deficiency, with approximately 42% of the population deemed to be at risk of inadequate zinc intake [39]. Thus, inadequate dietary intake of zinc is still a public health issue in the country, where zinc deficiency appears to be relatively common and an issue, in particular, for pregnant and lactating women, infants, and children [38, 40]. Although in humans, the prenatal effect of zinc deficiency on the risk of cardiometabolic disease in later life is less well studied, prenatal zinc supplementation has been shown to reduce the risk of microalbuminuria, metabolic syndrome, and peripheral adiposity in school-age offspring [41, 42].

Higher levels of LINE-1 methylation in adults have previously been associated with less body fat [43], a healthier lipid profile [44], and greater responses to weight loss programs [45]. Thus, zinc deficiency in the early stages of life may be a possible mechanism linking DNA methylation patterns and cardiometabolic phenotype in adulthood. For example, clinical studies have shown that zinc plays a role in regulating blood pressure and may be involved in the pathogenesis of hypertension [46, 47], with low levels of zinc in nulliparous women reported to be associated with an increased likelihood of gestational hypertension [48]. We observed that maternal zinc reduction was associated with lower birth weight and length. While in our study we did not observe any associations between maternal zinc levels and cardiometabolic parameters in young adult offspring, it may be that longer follow-up periods and larger sample sizes would be required to detect these. We did, however, detect novel associations between Alu and LINE-1 methylation patterns and cardiometabolic outcomes in offspring, namely total cholesterol levels and diastolic blood pressure, respectively. Of note, we observed that lower maternal zinc levels in late pregnancy were associated with increased LINE-1 partial methylation (uCmC) levels in the young adult offspring, which in turn, were associated with increased diastolic blood pressure. These findings appear to corroborate, for example, evidence from animal models showing that a maternal diet low in zinc was associated with impaired nephrogenesis and increased blood pressure in the offspring [49]. Thus, these results support potential associations between maternal zinc levels and developmental ’programming’ of long-term health through epigenetic changes in the offspring. However, even if confirmed by other studies, it remains unclear whether our observations would eventually be translated into overt metabolic disorders. As a result, our findings require corroboration by larger studies, preferably including older subjects.

Finding an association between maternal zinc status and LINE-1 methylation but not Alu methylation levels is not unique to our study. While both LINE-1 and Alu provide a proxy for global methylation levels, previous studies have found that their methylation levels do not always correlate and can vary by cell type, age, and gender [50–52]. Even within LINE-1 and Alu loci, DNA methylation is not always homogenous, so studying specific methylation patterns can be more informative than focusing solely on total LINE-1 and Alu methylation levels. For example, both hypo and hypermethylation LINE-1 loci were found in oral epithelial cells exposed to smoking [53]. Similarly, in keratinocytes, the pattern of LINE-1 methylation (mCmC, mCuC, uCmC) was more strongly associated with lichen simplex chronicus (neurodermatitis) than overall LINE-1 methylation [54]. Although LINE-1 and Alu have been used as surrogate markers for global DNA methylation in epigenetic epidemiology studies, their influence on gene expression can be wide-ranging given their location in non-coding regions of the genome. Other techniques, such as epigenome-wide association studies that identify differentially methylated regions within specific genes of interest (i.e., genes that by virtue of the protein products and pathways they are involved in have biological plausibility for outcomes studies) could better discern the associations between maternal zinc status, changes in DNA methylation in the offspring, and the phenotypic outcomes explored in this study [55].

Zinc deficiency has also been reported to be associated with poor fetal growth [2]. In our study, although no differences were observed in stratified analyses, lower maternal zinc levels at the end of pregnancy were associated with decreasing birth weight and length z-scores. These findings corroborate previous studies regarding birth weight [39, 56] and birth length [57]. Dietary zinc requirements during pregnancy are greater than in non-pregnant women to support fetal growth [36]. The mechanisms underpinning the association between zinc deficiency and fetal growth, however, are not fully understood but are likely a result of abnormal synthesis of nucleic acids, proteins, and cellular growth [2].

Our study had some limitations. Zinc methylation levels were only measured in a relatively small proportion of the follow-up study population, which might have contributed to the lack of observed associations with anthropometric and cardiometabolic outcomes. Zinc levels at earlier time-points during pregnancy were also largely unavailable, and the maternal zinc levels reported here might not be representative of maternal status earlier in pregnancy, as zinc concentrations decline throughout pregnancy [36]. Further, the offspring were assessed at ≈20 years of age and might have been too young to display overt signs of adverse cardiometabolic outcomes. Lastly, their lifestyle characteristics (i.e., diet and physical activity) could have mitigated potential adverse outcomes, but such data were not collected on our study participants. Nonetheless, this study provides novel data comparing maternal zinc concentrations during pregnancy and methylation levels in the young adult offspring. These associations have never been examined in Thailand, and rarely elsewhere.

## Conclusions

This study showed for the first time that lower zinc concentrations in maternal plasma at the end of pregnancy were associated with decreased LINE-1 methylation in the young adult offspring. Thus, epigenetic modifications as a result of exposure to a zinc-deficient intrauterine milieu may lead to long-term phenotypic changes in the offspring, although the consequences to offspring health are still unclear. Similarly, the mechanism(s) by which zinc influences DNA methylation patterns are yet to be elucidated. Furthermore, in this context, the effects of prenatal zinc supplementation on DNA methylation in offspring warrants further research, particularly in populations with a high prevalence of zinc deficiency.

## Supporting information

S1 Fig Distribution of maternal zinc concentrations in late pregnancy according to group stratification. (PDF)

S1 Table Demographic and birth characteristics of study participants stratified into tertiles of maternal plasma zinc concentrations in late pregnancy. (PDF)

S2 Table Linear associations between LINE-1 methylation and anthropometric and cardiometabolic outcomes in young adult offspring. (PDF)

S3 Table Linear associations between Alu methylation and anthropometric and cardiometabolic outcomes in young adult offspring. (PDF)
