# Supplementary material for: Associations between maternal plasma zinc concentrations in late pregnancy and LINE-1 and Alu methylation loci in the young adult offspring
Source: PLoS One. 2022 Dec 30;17(12):e0279630. doi: 10.1371/journal.pone.0279630 (PMC9803117; doi:10.1371/journal.pone.0279630)
Supplement: S1 Fig — (PDF) [file pone.0279630.s001.pdf]

S1 Figure

Distribution of maternal zinc concentrations in late pregnancy according to group stratification.

A) Mothers with zinc deficiency (plasma level <50 µg/dL; *n*=39; red) vs those without zinc deficiency (≥50 µg/dL; *n*=35; blue). Horizontal bars represent the quartile 1, median, and quartile 3.

B) Tertiles stratified according to maternal zinc concentrations in plasma as: Lower (<41.0 µg/dL; *n*=24), Mid (≥41.0 but <58.05 µg/dL; *n*=26), and Higher (≥58.05 µg/dL; *n*=24). Horizontal bars represent the quartile 1, median, and quartile 3.

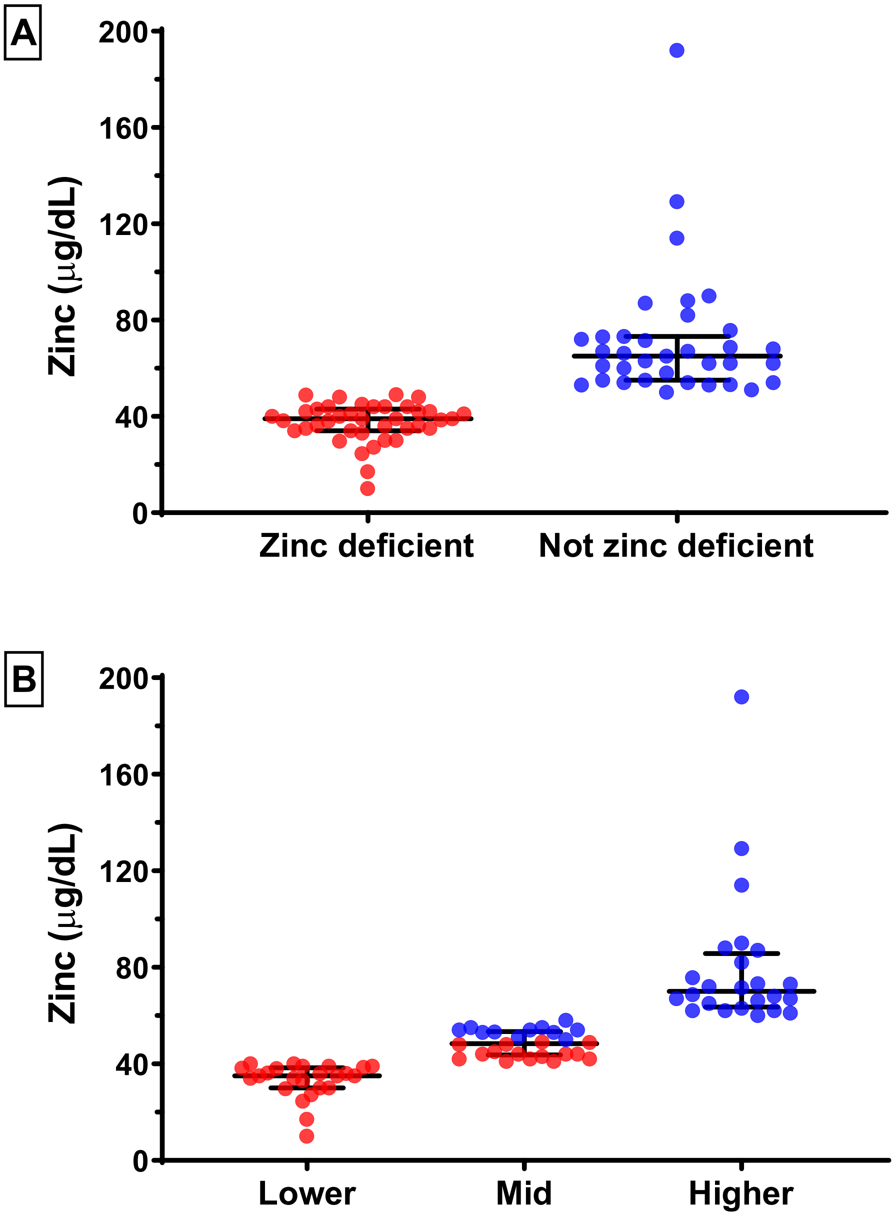

Rerkasem A, Nantakool S, Wilson BV, Mangklabruks A, Boonyapranai K, Mutirangura A, Derraik JGB, Rerkasem K. Associations between maternal plasma zinc concentrations in late pregnancy and LINE-1 and Alu methylation loci in the young adult offspring. PLoS ONE.
