# Supplementary material for: Associations between maternal plasma zinc concentrations in late pregnancy and LINE-1 and Alu methylation loci in the young adult offspring
Source: PLoS One. 2022 Dec 30;17(12):e0279630. doi: 10.1371/journal.pone.0279630 (PMC9803117; doi:10.1371/journal.pone.0279630)
Supplement: S1 Table — (PDF) [file pone.0279630.s002.pdf]

S1 Table

Demographic and birth characteristics of study participants stratified into tertiles of maternal plasma zinc concentrations in late pregnancy.

Participants	Characteristics	Lower	Mid	Higher	*p*-value
n		24	26	24	
**Mother (at baseline)**	**Age (years)**	25.3 ± 4.7	27.7 ± 4.8	25.4 ± 3.2	0.09
	**Maternal body mass index (kg/m^2)**	22.02 ± 2.67	21.42 ± 3.06	20.99 ± 2.11	0.40
	**Nulliparous**	15 (63%)	15 (58%)	20 (83%)	0.12
	**Pregnancy-induced hypertension [a]**	2 (8%)	3 (12%)	nil	0.36
	**Education level**				
	Less than high school	23 (96%)	22 (92%)	20 (95%)	0.54
	High school or higher	1 (4%)	2 (8%)	1 (5%)	
	**Household income (baht/month) [b]**	2808 [1900, 4525]	2200 [1475, 3650]	3300 [1000, 4800]	0.38
**Offspring (at birth)**	**Sex (male)**	8 (33%)	13 (50%)	11 (46%)	0.49
	**Gestational age (weeks)**	39.5 ± 1.6	38.7 ± 1.6	39.0 ± 1.6	0.34
	**Caesarean delivery**	3 (13%)	1 (4%)	1 (4%)	0.52
	**Preterm birth [c]**	2 (8%)	3 (12%)	2 (8%)	>0.99
	**Birth weight (g)**	2801 ± 409	2665 ± 329	2836 ± 327	0.21
	**Birth weight *z*-score**	-1.05 ± 0.96	-1.14 ± 0.69	-0.82 ± 0.79	0.38
	**Low birth weight (<2,500 g)**	6 (25%)	4 (15%)	3 (13%)	0.55
	**Birth length (cm)**	47.7 ± 2.2	47.5 ± 2.0	48.7 ± 2.2	0.15
	**Birth length *z*-score**	-0.78 ± 1.25	-0.68 ± 0.97	-0.08 ± 1.21	0.11
**Offspring (at follow-up)**	**Age (years)**	20.7 ± 0.5	20.7 ± 0.4	20.4 ± 0.5	0.11
	**Body mass index (kg/m^2)**	20.95 ± 4.62	20.47 ± 3.32	21.15 ± 3.03	0.80
	**Smoking status**				
	Non-smoker	22 (100%)	23 (92%)	16 (84%)	0.21
	Smoker	nil	2 (8%)	3 (16%)	
	**Education**				
	Less than high school	4 (21%)	2 (9%)	3 (16%)	0.54
	High school or higher	15 (79%)	21 (91%)	16 (84%)	

Data are means ± standard deviation (SD), medians [quartile 1, quartile 3], or n (%), as appropriate
[a] Pregnancy-induced hypertension was defined as systolic blood pressure ≥140 mmHg and/or diastolic blood pressure ≥90 mmHg during pregnancy, which was developed after 20 weeks of gestation without proteinuria, in a woman previously normotensive.
[b] Income at recruitment to the original study in 1989–1990, unadjusted for inflation.
[c] Defined as gestational age at birth <37 weeks.
There was no reported use of tobacco or illicit drugs during pregnancy.
