# Supplementary material for: Associations between maternal plasma zinc concentrations in late pregnancy and LINE-1 and Alu methylation loci in the young adult offspring
Source: PLoS One. 2022 Dec 30;17(12):e0279630. doi: 10.1371/journal.pone.0279630 (PMC9803117; doi:10.1371/journal.pone.0279630)
Supplement: S2 Table — (PDF) [file pone.0279630.s003.pdf]

S2 Table

Linear associations between LINE-1 methylation and anthropometric and cardiometabolic outcomes in young adult offspring.

		n	Total LINE-1 (%)	LINE-1 mCmC (%)	LINE-1 mCuC (%)	LINE-1 uCmC (%)	LINE-1 uCuC (%)
**Anthropometry**	**Height (cm)**	64	0.9 (-1.7, 3.5)	0.4 (-0.9, 1.7)	-1.0 (-2.5, 0.5)	0.2 (-0.9, 1.3)	-0.1 (-2.6, 2.4)
	**Weight (kg)**	64	1.59 (-3.41, 6.59)	0.89 (-1.62, 3.40)	0.00 (-2.93, 3.00)	-0.62 (-2.74, 1.50)	-0.10 (-4.93, 4.74)
	**BMI (kg/m^2)**	64	0.34 (-1.21, 1.89)	0.22 (-0.56, 1.00)	0.27 (-0.64, 1.18)	-0.30 (-0.95, 0.35)	0.01 (-1.48, 1.51)
**Lipid profile**	**Total cholesterol (mg/dL)**	62	11.6 (-10.9, 34.1)	7.4 (-3.6, 18.4)	-6.6 (-18.6, 5.4)	-2.5 (-11.4, 6.5)	8.7 (-14.5, 32.0)
	**HDL-C (mg/dL)**	64	-2.0 (-8.4, 4.3)	-0.3 (-3.5, 2.9)	-0.1 (-3.8, 3.7)	-0.3 (-3.0, 2.4)	2.8 (-3.2, 9.0)
	**LDL-C (mg/dL)**	62	10.6 (-9.2, 30.5)	6.4 (-3.3, 16.1)	-6.5 (-17.1, 4.1)	-1.4 (-9.3, 6.5)	5.8 (-14.7, 26.4)
	**Triglycerides (mg/dL)**	64	8.2 (-13.5, 30.0)	4.6 (-6.2, 15.5)	-0.5 (-13.3, 12.3)	-4.2 (-13.4, 4.9)	6.2 (-14.7, 27.2)
**Blood pressure**	**Systolic (mmHg)**	64	-1.8 (-6.1, 2.5)	-0.7 (-2.9, 1.5)	-1.7 (-4.2, 0.8)	1.0 (-0.9, 2.8)	2.3 (-1.9, 6.4)
	**Diastolic (mmHg)**	64	-0.5 (-5.5, 4.6)	-0.9 (-3.4, 1.6)	-2.4 (-5.3, 0.6)	**2.4 (0.3, 4.4)***	-2.6 (-7.4, 2.3)
**Glucose metabolism**	**Fasting glucose (mg/dL)**	63	-2.38 (-6.16, 1.40)	-1.12 (-3.02, 0.77)	0.02 (-2.27, 2.31)	0.47 (-1.14, 2.08)	1.66 (-1.98, 5.31)
	**HOMA-IR**	63	0.60 (-0.23, 1.43)	0.26 (-0.16, 0.67)	-0.30 (-0.79, 0.20)	0.00 (-0.36, 0.35)	-0.15 (-0.95, 0.66)

BMI, body mass index; HDL-C, high-density lipoprotein cholesterol; HOMA-IR, homeostatic model assessment of insulin resistance; and LDL-C, low-density lipoprotein cholesterol.
CpG methylation patterns include: mCmC, hypermethylation; uCuC, hypomethylation; mCuC and uCmC, partial methylation.
Data are the adjusted β coefficients and 95% confidence intervals from general linear models, adjusted for sex, maternal age at baseline, and gestational age, with other independent variables added where appropriate: maternal BMI for offspring weight and BMI; maternal height for offspring height; and pregnancy-induced hypertension and current smoking status for blood pressure. Note that the adjusted β coefficients represent the change in outcome for every 10 percentage points increase in methylation levels. *p<0.05 for a statistically significant association (shown in bold) between LINE-1 methylation and a given offspring outcome.
