# Supplementary material for: Associations between maternal plasma zinc concentrations in late pregnancy and LINE-1 and Alu methylation loci in the young adult offspring
Source: PLoS One. 2022 Dec 30;17(12):e0279630. doi: 10.1371/journal.pone.0279630 (PMC9803117; doi:10.1371/journal.pone.0279630)
Supplement: S3 Table — (PDF) [file pone.0279630.s004.pdf]

S3 Table 3

Linear associations between Alu methylation and anthropometric and cardiometabolic outcomes in young adult offspring.

		n	Total Alu (%)	Alu mCmC (%)	Alu mCuC (%)	Alu uCmC (%)	Alu uCuC (%)
**Anthropometry**	**Height (cm)**	72	-0.3 (-2.4, 1.7)	-0.9 (-2.7, 0.9)	1.7 (-0.7, 4.1)	0.5 (-1.6, 2.7)	-0.3 (-1.8, 1.3)
	**Weight (kg)**	72	-1.47 (-5.68, 2.75)	-3.41 (-7.09, 0.27)	4.17 (-0.84, 9.18)	3.13 (-1.26, 7.52)	-0.81 (-4.04, 2.43)
	**BMI (kg/m^2)**	72	-0.49 (-1.93, 0.95)	-1.04 (-2.31, 0.23)	1.11 (-0.62, 2.84)	1.07 (-0.43, 2.57)	-0.22 (-1.33, 0.89)
**Lipid profile**	**Total cholesterol (mg/dL)**	69	9.3 (-8.3, 26.8)	-7.3 (-23.7, 9.0)	17.1 (-3.0, 37.3)	**20.6 (4.0, 37.3)***	**-13.6 (-25.9, -1.3)***
	**HDL-C (mg/dL)**	72	2.4 (-2.3, 7.2)	0.2 (-4.0, 4.4)	1.0 (-4.8, 6.7)	4.4 (-0.6, 9.3)	-2.8 (-6.4, 0.8)
	**LDL-C (mg/dL)**	69	7.4 (-7.9, 22.8)	-5.6 (-19.8, 8.7)	15.3 (-2.2, 32.8)	14.2 (-0.6, 29.0)	-10.6 (-21.4, 0.21)
	**Triglycerides (mg/dL)**	72	-1.9 (-20.5, 16.7)	-7.0 (-23.0, 9.0)	8.1 (-13.8, 30.9)	9.9 (-9.6, 29.3)	-3.2 (-17.4, 11.0)
**Blood pressure**	**Systolic (mmHg)**	72	2.4 (-1.1, 5.8)	0.5 (-2.4, 3.4)	0.7 (-3.4, 4.7)	3.0 (-0.5, 6.6)	-2.4 (-5.0, 0.3)
	**Diastolic (mmHg)**	72	1.9 (-2.1, 5.9)	1.9 (-1.5, 5.2)	-1.2 (-5.8, 3.4)	-0.7 (-4.9, 3.4)	-0.6 (-3.8, 2.4)
**Glucose metabolism**	**Fasting glucose (mg/dL)**	71	-2.44 (-5.31, 0.43)	-1.63 (-4.21, 0.96)	0.85 (-2.68, 4.38)	-1.99 (-5.06, 1.08)	1.76 (-0.43, 3.95)
	**HOMA-IR**	71	0.02 (-0.69, 0.65)	-0.34 (-0.93, 0.25)	0.47 (-0.33, 1.27)	0.43 (-0.27, 1.14)	-0.02 (-0.71, 0.31)

BMI, body mass index; HDL-C, high-density lipoprotein cholesterol; HOMA-IR, homeostatic model assessment of insulin resistance; and LDL-C, low-density lipoprotein cholesterol.
CpG methylation patterns include: mCmC, hypermethylation; uCuC, hypomethylation; mCuC and uCmC, partial methylation.
Data are the adjusted β coefficients and 95% confidence intervals from general linear models, adjusted for sex, maternal age at baseline, and gestational age, with other independent variables added where appropriate: maternal BMI for offspring weight and BMI; maternal height for offspring height; and pregnancy-induced hypertension and current smoking status for blood pressure.
Note that the adjusted β coefficients represent the change in outcome for every 10 percentage points increase in methylation levels. *p<0.05 for a statistically significant association (shown in bold) between Alu methylation and a given offspring outcome.

Rerkasem A, Nantakool S, Wilson BV, Mangklabruks A, Boonyapranai K, Mutirangura A, Derraik JGB, Rerkasem K. Associations between maternal plasma zinc concentrations in late pregnancy and LINE-1 and Alu methylation loci in the young adult offspring. PLoS ONE.
